# Innovating physical education with artificial intelligence: a potential approach

**DOI:** 10.3389/fpsyg.2025.1490966

**Published:** 2025-04-10

**Authors:** Bin Cui, Wei Jiao, Shuying Gui, Yang Li, Qun Fang

**Affiliations:** ^1^Faculty of Physical Education, Shanghai University of Medicine & Health Sciences, Shanghai, China; ^2^Teaching Group of Physical Education, Shanghai Qibao High School, Shanghai, China; ^3^Physical Education Department, Shanghai University of Finance and Economics, Shanghai, China; ^4^School of Physical Education, Qingdao University, Qingdao, China

**Keywords:** physical education, artificial intelligence, health promotion, education innovation, closed-loop design

## Introduction

Physical Education (PE) is widely regarded as a critical and efficient way to promote health for children and adolescents through physical activity. According to the World Health Organization (WHO), people at this age group are recommended to spend at least 60 min of moderate to vigorous daily physical activity (World Health Organization, 2020). Nevertheless, very few children and adolescents meet this recommendation, a disappointing feature according to Bull et al. (2020). PE significantly contributes to promoting health standards and increasing physical activities among children and adolescents. Due to this subject, students have chances to perform sports skills, engage in repetitive practice, participate in competitions, and get scientific assessments, all of which are essential components of the PE process. In recent years, the public has paid much more attention to the overall enhancement of PE. Several factors including teacher professionality, facility conditions, and curriculum types interact in determining the extent with which moderate-to-vigorous physical activity (MVPA) may take place during a PE period (Bevans et al., 2010). More generally, the collaboration and coordination between community, family, and school-based interventions is a real-world issue of concern, deserving attention and deliberation (Tilga et al., 2023).

China, as the pioneer in implementing physical fitness monitoring for children and adolescents, continues to face challenges throughout the entire process of PE (Brazo-Sayavera et al., 2024). These challenges persist and impact various aspects of PE. One of the primary challenges is the large class sizes and limited availability of suitable facilities. With crowded classrooms and inadequate sports facilities, it becomes difficult to provide high-quality PE classes. Additionally, PE teachers often have a heavy workload, resulting in a decline in the overall quality of instruction. Another challenge lies in the types of PE classes offered. The effects of the “mindless” aerobic exercise alone are limited. When children and adolescents love certain activities and are motivated, they will participate more and feel positive emotions (Diamond, 2015). It is crucial to explore innovative teaching approaches and instructional strategies that can reignite students' enthusiasm for PE. Multicomponent interventions involving the family or community in addition to the school are likely to be most effective (Sluijs et al., 2007; Brown et al., 2017), but physical activity homework is relatively underutilized (Duncan et al., 2019). Moreover, the pressure associated with data collection, analysis, and utilization presents challenges to the overall PE process. The collection and management of data require resources and expertise, posing additional burdens on teachers and administrators.

Due to the huge number of students and a shortage of human resources such as teachers and competent instructors, it has been a long-lasting difficulty to have a simple and efficient PE process. Thus, it is imperative to improve teaching efficiency and effectively integrate various teaching systems. In this regard, Artificial Intelligence (AI) comes at this juncture as an innovative option. With the power of AI, we can explore new approaches to enhancing PE, such as personalized instruction, data analysis, and interactive learning tools.

According to the theory of PE control, the attainment of educational objectives in the realm of PE necessitates not only the establishment of clear goals but also the implementation of systematic control mechanisms throughout the PE process. Effectively reaching any desired outcome is contingent upon the closed-loop design and strategic regulation of the involved processes, thereby facilitating more efficient and timely achievement. A study highlights that effective process control in PE can significantly enhance students' health levels, promote their physical development, and foster a healthy lifestyle (Zhamardiy et al., 2019).

In PE, through real-time evaluation of students' practice process, teachers can promptly identify students' deficiencies in skill mastery and make targeted adjustments, thereby improving students' overall performance (Haible et al., 2019). Process control can also help educators identify and solve potential problems in the educational process, thereby optimizing teaching strategies and improving educational quality (Gallotta et al., 2024). Building on this theoretical framework, the integration of AI within academic PE can be conceptualized as the systematic development of a closed-loop scheme, accomplished through the utilization of machine learning algorithms and multimodal perception technologies. This framework encompasses several critical components—including data collection, dynamic modeling, teaching feedback, and practical intervention.

As an advanced data-driven technology, AI has witnessed impressive progress since the late 1990s. This is due to the advent of machine learning and deep learning methods, as well as a dramatic increase in computational power and increased digitization in all domains (Mccorduck, 2004). AI has penetrated all fields in unprecedented and seemingly impossible ways and also exerted substantial impacts on sports (Araújo et al., 2021). Information and communication technologies have opened the digital environment, improved accessibility in PE (Wang and Park, 2021), and greatly impact the way PE are taught nowadays (Legrain et al., 2015). In sports, an information system can be established upon a series of interactive components. The environment processes sensory inputs including heart rate measurements, image data, and even complete video streams. Cognitive models are established to predict the quality of exercise regimen outcomes. Action plans are searched and optimized by means of feedback from users to implement resulting policies to act on or interact with the environment (Hammes et al., 2022).

## AI as a key technology: efficient connection of PE processes

Image recognition and computer vision techniques are extensively applied in recognizing movements. Recent technological advances support motion track through portable device. Computer vision can use specially trained machine learning models to identify human joints in real-time video, enabling label-free motion capture (Khanal et al., 2022). At present, infrared sensor equipment is often used for data collection and testing in PE. However, the equipment is easily disturbed by environmental backgrounds (Qu et al., 2017). It is difficult to realize the evaluation of technical action mode in outdoor environment. Shifting from traditional ways of collecting via sensors to a vision-based data acquisition is becoming increasingly important. Computer vision technology possesses huge potential to be applied in PE. Compared with conventional laboratory equipment, it can achieve marker-free object or body tracking more easily. As shown by recent research, combining computer vision technology with marker-free motion capture stands as an efficient method of automatically detecting human movements in racquet, weightlifting, and other sports (Novatchkov and Baca, 2013; Fernandez et al., 2022; Tan et al., 2024).

In recent years, development of hardware and software, along with improved computational power and efficiency, has led to the emergence of open-source pose estimation algorithms (Colyer et al., 2018). These algorithms work with video data captured from a single camera device, thus eliminating the use of complicated setup and enabling data collection from various PE settings (Mathis et al., 2018). The algorithm worked very well against marker-based motion capture with minimal training data and was able to track test frames to a high degree of accuracy (Colyer et al., 2018; Mathis et al., 2018). It has been demonstrated that low-cost 2D video analysis, when combined with computer vision technology but without the requirement for marker, is capable of producing an accurate analysis of running technology (Hooren et al., 2023). Another study indicated that marker-free systems are performed similarly to marker-based systems, and the use of the former can now be extended to attempt effective dynamic motion performance monitoring in football (Aughey et al., 2022).

In addition, designs based on machine learning methods can accomplish classification, pattern recognition, and prediction of specific motion data, especially through self-learning algorithms such as artificial neural networks for performance analysis in sports activities related to mathematics and computer science (Molavian et al., 2023). There exist several successful implementations of such technology, such as analysis and research on different sports evaluations in baseball and basketball (Ghasemzadeh and Jafari, 2011; Lamb et al., 2010). AI-based technologies such as SVM have proved to be influential tools in data learning and solving classification and regression problems with superior classification performance (Wang et al., 2022). Furthermore, SVM can execute the built-in classification of runway inclination and velocity parameters (Eskofier et al., 2010) or identify differences in kinematic features (Fischer et al., 2011). A recent study on the economics of running identified variables, and the proposed method will allow professionals to analyze practitioners' performance in detail and improve their performance by observing measured force and displacement time series or computing characteristics such as acceleration, velocity, and power in real time (Hooren et al., 2024).

Identification, acquisition, and collection of clusters in sport-specific data allow appropriate recommendations of sports activities in terms of the abilities of persons with specific demands. According to the research on AI-driven digital physical activity interventions for older adults, as time goes by, personalized feedback about completed exercises, overall health status, mobility patterns, and other trends can be used as positive reinforcement to enhance continued engagement in these types of digital program (Wong et al., 2023). There are many types of digital sporting interventions. These range from digital platforms offering streaming workout videos and interactive sports apps based on wearable technologies that capture and promote physical activity specific to a diverse aging population to advanced applications of augmented and virtual realities offering semi-digital and fully digital contexts improving exercise experiences (O'Connor, 2019; Choi et al., 2021).

AI has combined large-scale data analysis with machine learning to provide tailored advice in sports settings. In this respect, several studies have shown that OpenAI's GPT-4 can indeed contribute to safe exercising through valuable suggestions. In sports, ChatGPT can customize training prescriptions based on specified information, including plans, recommendations, and performance feedback (Dergaa et al., 2023a). It is worth noting that ChatGPT cannot replace judgment and experience of human practitioners (Washif et al., 2023), enhancing real-time interaction, improving personalization and adaptive algorithms, and achieving variability in exercises are directions that need to be advanced in the future (Dergaa et al., 2023b).

The existing literature shows that the development of low-cost image recognition and computer vision technology in the field of sports, the advancement of hardware and software technology, and the improvement of computing power and efficiency provide better help for the efficient development of PE. In the current wave of PE reform, AI as a key technology, is increasingly being applied to various educational processes to achieve efficient connectivity in the PE process. Through data analysis and intelligent algorithms, AI can optimize course design, teaching management, and evaluation, promote efficient flow and interaction of information, and thus improve overall teaching effectiveness. By introducing the control concept of closed-loop teaching design into PE, we can integrate the implementation of various stages of teaching, enabling teaching strategies to be adjusted in real time according to students' performance and needs, thus achieving a more flexible and efficient educational model.

## Closed-loop design based on PE process

**Figure 1:**
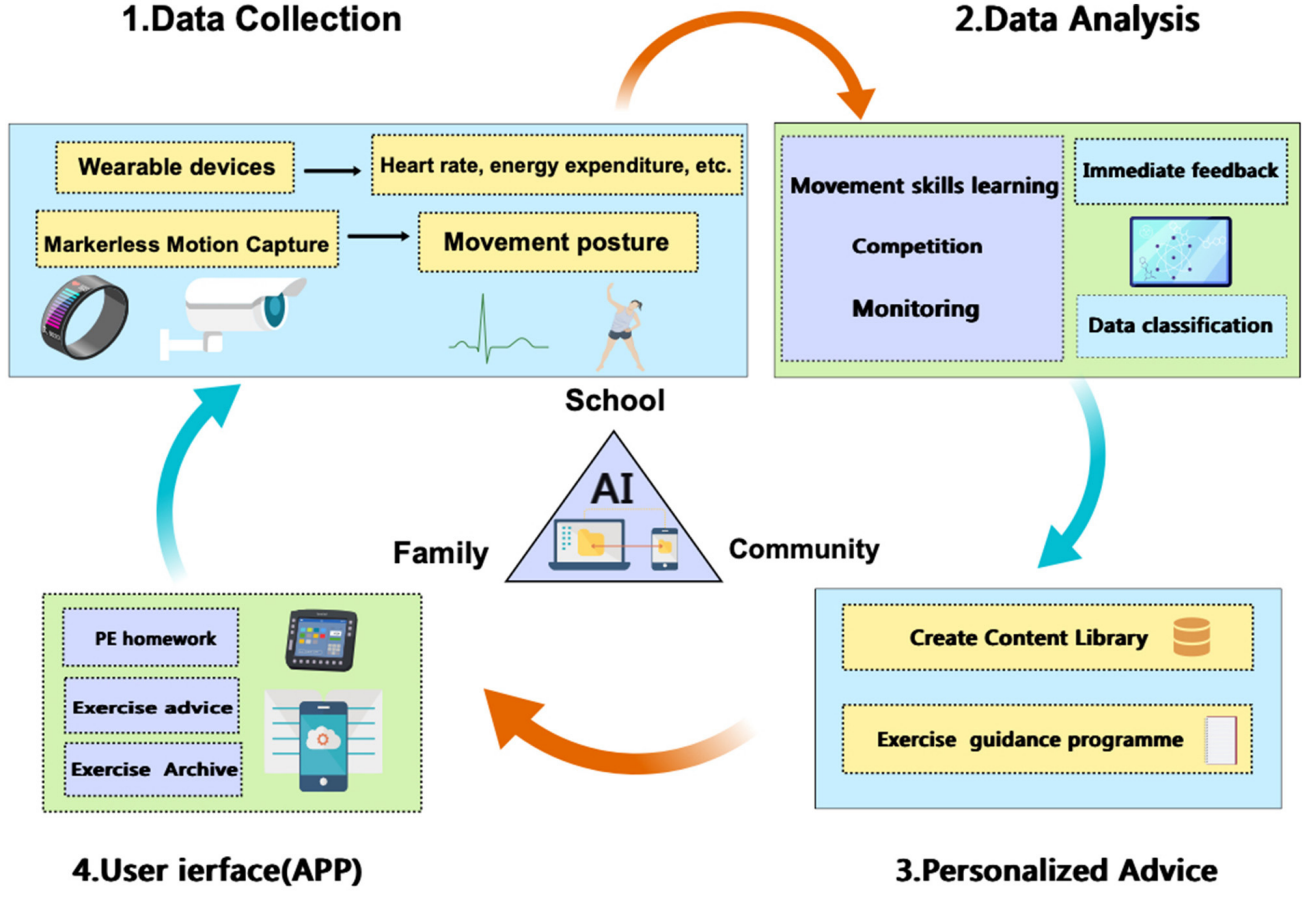
Framework of AI-based PE (created with MedPeer).

Under the background of the rise of AI, researchers call for the design of an innovative integrated feedback closed-loop scheme of AI + PE, so as to improve the physical health of children and adolescents. The closed-loop design requires integration to complete exercises, competitions, and tests in high accuracy and efficiency (Figure 1). On the one hand, it aims to help students improve their health status through data collection and testing, behavior pattern feature recognition and interest-based (such as VR, AR, etc.) (Noury et al., 2022) intervention, and personalized optimization. On the other hand, it realizes multi-platform sharing of school, community, and family sports through AI to form synergy. Specifically, several key links are required:

Use smart devices (such as wearables, smartphone apps, health monitors, etc.) to track and record biological data, including heart rate, activity, energy expenditure (Thiebaud et al., 2018), or motion data captured through marker-free motion capture, such as movement posture, height, and movement angle. The motion capture technology based on computer vision has made significant progress in recognition accuracy and reliability, making it applicable to various sports scenes (Suo et al., 2024). Subsequently, machine algorithms or deep learning are used to model and analyze the collected data to realize the recognition and prediction of children and adolescents' health patterns and action characteristics, and to classify user behaviors. Design a teaching intervention and feedback mechanism for human-computer interaction that leverages gamified scenarios or digital tools such as VR and AR. This mechanism should analyze user data through AI to provide immediate corrections and suggestions. When the user completes a certain healthy behavior, the system gives positive feedback in time, such as virtual rewards, points, etc. Simultaneously, introducing social elements to encourage users to share their health achievements and increase motivation.

According to the data analysis results, the AI generates personalized health advice on various aspects (Shaban-Nejad et al., 2018), including diet plans, exercise guidance, schedule adjustments, etc. Provide goal setting functions, such as setting weight loss goals, muscle gain goals or improving physical fitness, and dynamically adjust suggestions according to user progress. Construct a content library, grading practice content, ensuring diversified content suitable for practitioners of different skill levels, regularly updating content library, and maintaining updates and relevance of materials.

A simple and interactive interface should be developed for school administrators, teachers, parents, and community instructors of different ages based on computer cloud and mobile app design, enabling users to easily understand student health data and get advice, and track student progress and performance. The system is also expected to provide teaching guides and advice to help community mentors, parents, coach, and students to complete sports homework. Finally, it is important to ensure that all personal data are encrypted. Users have complete control over sharing and use of data.

## Conclusion

China, as one of the earliest nations to focus on monitoring the physical fitness of adolescents, continues to face challenges in achieving overall efficiency in PE. The large class sizes and high number of weekly classes for PE teachers result in declined quality of PE class, decreasing students' interest, and engagement. The need for real-time feedback, data collection, analysis, and personalized suggestions based on individual characteristics remains ongoing issues. In addition, school PE and home-based interventions synergize to provide a promising avenue to address the pandemic of physical inactivity among adolescents. AI has gradually penetrated all fields of sports, and the field of PE should not be an exception. Low-cost investment improves the efficiency of practice, competition, monitoring, and other links, saving human resources. AI helps students improve their health through data collection and testing, motion pattern recognition, and personalized optimization. At the same time, AI can facilitate the sharing of multiple platforms for school-based and family PE, allowing for collaboration and synergy. Therefore, AI can serve as an efficient way to promote the connectivity of PE.
